# Transcriptome exploration of ferroptosis-related genes in TGFβ- induced lens epithelial to mesenchymal transition during posterior capsular opacification development

**DOI:** 10.1186/s12864-024-10244-y

**Published:** 2024-04-09

**Authors:** Cong Fan, Chao Wang, Yan Wang, Jian Jiang

**Affiliations:** 1 Eye Center of Xiangya Hospital, Central South University, Changsha, China; 2 Hunan Key Laboratory of Ophthalmology, Xiangya Hospital, Central South University, Changsha, China; 3 National Clinical Research Center for Geriatric Disorders, Xiangya Hospital, Central South University, Changsha, China; 4 Department of Biological Sciences, University of Delaware, Newark, USA

**Keywords:** RNA-sequencing, Transforming growth factor beta, Primary lens epithelial cells, Posterior capsular opacification, Epithelial-mesenchymal transition

## Abstract

**Background:**

Posterior capsular opacification (PCO) is the main reason affecting the long-term postoperative result of cataract patient, and it is well accepted that fibrotic PCO is driven by transforming growth factor beta (TGFβ) signaling. Ferroptosis, closely related to various ocular diseases, but has not been explored in PCO.

**Methods:**

RNA sequencing (RNA-seq) was performed on both TGF-β2 treated and untreated primary lens epithelial cells (pLECs). Differentially expressed genes (DEGs) associated with ferroptosis were analyzed using Gene Ontology (GO) and Kyoto Encyclopedia of Genes and Genomes (KEGG) to investigate their biological function. Additionally, protein-to-protein interactions among selected ferroptosis-related genes by PPI network and the top 10 genes with the highest score (MCC algorithm) were selected as the hub genes. The top 20 genes with significant fold change values were validated using quantitative real-time polymerase chain reaction (qRT-PCR).

**Results:**

Our analysis revealed 1253 DEGs between TGF-β2 treated and untreated pLECs, uncovering 38 ferroptosis-related genes between two groups. Among these 38 ferroptosis-related genes,the most prominent GO enrichment analysis process involved in the response to oxidative stress (BPs), apical part of cell (CCs),antioxidant activity (MFs). KEGG were mainly concentrated in fluid shear stress and atherosclerosis, IL-17 and TNF signaling pathways, and validation of top 20 genes with significant fold change value were consistent with RNA-seq.

**Conclusions:**

Our RNA-Seq data identified 38 ferroptosis-related genes in TGF-β2 treated and untreated pLECs, which is the first observation of ferroptosis related genes in primary human lens epithelial cells under TGF-β2 stimulation.

**Graphical Abstract:**

Our study initially observed ferroptosis related genes in primary human lens epithelial cells stimulated by TGF-β2. These findings may improve the understanding of the molecular mechanisms of PCO and provide a new direction for exploring the potential mechanisms of PCO

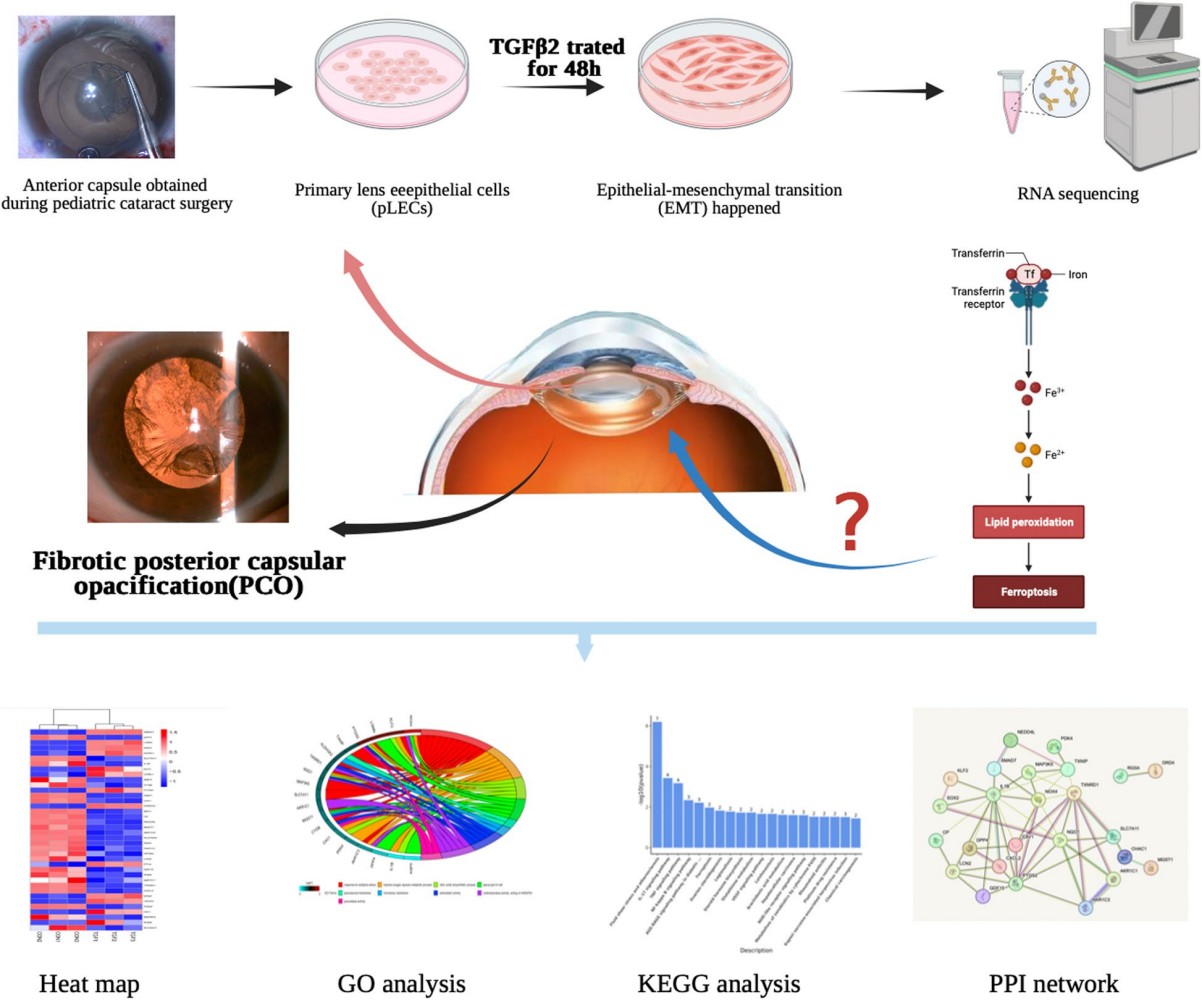

**Supplementary Information:**

The online version contains supplementary material available at 10.1186/s12864-024-10244-y.

## Introduction

Cataract is the leading blind-causing disease in the world, and the prevalence rate of cataract is 17.2% worldwide, which is the primary reason of blindness aged 50 and above [1, 2]. Its implications extend beyond vision impairment, profoundly impacting both physical and mental health as well as quality of life [3]. At present, there is no specific medicine effectively treats cataract, and surgery is still the most viable option [4]. Posterior capsular opacification (PCO) is a main reason affecting the long-term postoperative result of cataract patients. It’s been documented that the prevalence of PCO in adults can escalate to 40% a decade after surgery, while in infants, the rate of PCO occurrence is nearly 100% just one-year after surgery [5, 6]. The treatment most often administered for PCO is Nd:YAG laser capsulotomy. However, this treatment is not without risks, as complications include retinal detachment, damage of the intraocular lens(IOL), macular cystoid edema, and increased intraocular pressure [7].After many times of laser treatment or even surgery, PCO can still recur after cataract surgery in children [6], which undoubtedly increases the risk of treatment and the financial burden on families. Therefore, the prevention of PCO is still a critical focus in cataract research, especially for cataract surgery in children. Ophthalmologists around the world are constantly exploring the strategies to prevent PCO.

The treatment for cataracts typically involves the surgical excision of the anterior capsule, which is attached to the lens epithelial cells (LECs) and lens fibers, while preserving the lens capsule to support IOL [8]. However, it is challenging to remove all LECs surgically, and the pathological process behind PCO involves the proliferation, migration, and epithelial-mesenchymal transition (EMT) of residual LECs, a mechanism that is not fully understood post-surgery [9]. Postoperative trauma and inflammatory reaction also contribute to this process. EMT induces the transformation of LECs into myofibroblasts, contributing to fibrotic PCO and anterior subcapsular cataract(ASC) [9]. Therefore, EMT is crucial process in the development of PCO, especially in fibrotic PCO.

Ferroptosis is a distinct type of cell death that relies on iron and significantly impacts various disorders such as cancers, neurodegenerative diseases, acute renal damage, and ischemia/reperfusion injuries [10]. The accumulation of ferrous iron (Fe2 +) and lipid peroxidation are the essential factors of ferroptosis onset [11]. Additionally, ferroptosis has a strong correlation with multiple eye diseases, such as keratopathy [12], glaucoma [13, 14], age-related macular degeneration [15], diabetic retinopathy [16]. However, research on the correlation between ferroptosis and cataract mainly focus on age-related cataract (ARC). It is widely believed that fibrotic PCO is driven by transforming growth factor beta (TGFβ) signaling, as active TGFβ is sufficient to induce the fibrotic response [17, 18]. Therefore, we used TGF-β2 to induce EMT processes in primary Lens Epithelial Cells(pLECs) to simulate the development of fibrotic PCO, which is a commonly used and well-established model.

In this study, TGF-β2 treated and untreated pLECs were collected for RNA sequencing to identify differentially expressed ferroptosis genes associated with fibrotic PCO. Our findings observed ferroptosis related genes in primary human lens epithelial cells under TGF-β2 stimulation, providing novel insights for preventing the occurrence of fibrotic PCO.

## Materials and methods

### Patient characteristics

Thirteen children (age 5 months to 8 years old) with binocular or monocular pediatric cataracts presenting to the Xiangya Hospital Eye Center, from January 2021 to December 2021 took part in this study and with the consent of their guardians. Ethical approval for the protocol has been obtained (grant number: 202112639).

### Cultivation of primary lens Epithelial cells(pLECs)

We gathered 20 anterior lens capsules from 13 patients during the curvilinear capsulorhexis stage of pediatric cataract surgery. Modifying the procedure outlined by Wernecke [19], we established the following pLECs cultivation protocol:

1. The anterior lens capsules that adhered to pLECs obtained during pediatric cataract surgery, were laid as flat as possible in a 6 cm culture well and fixed at the bottom of the well with a glass coverslip. They were subsequently cultured in Dulbecco's Modified Eagle Medium (DMEM) with high glucose (Hyclone, Thermo Scientific, USA,CAT#C11995500BT), enriched with 20% fetal bovine serum (FBS, Gibco, USA,CAT#10099141) and 100 U/ml penicillin and 100 μg/mL streptomycin mixture(NCM Biotech,China,CAT#C100C5) at 37°C in a humidified atmosphere containing 5% CO_2_.
2. The medium was replaced every three days once the cells began to migrate on the well’s bottom. Confluence was achieved at 7 to 15 days, observed as cells migrating on the bottom of the well and glass coverslip. pLECs were subcultured for 3 to 4 generations and seeded on 6-well plate.
3. Upon achieving a confluence of 70%, the pLECs were treated with 5 ng/mL TGF-β2 (Proteintech, USA,CAT#HZ-1092) for a period of 48 hours in a serum-free medium.

### Cell line culture

The SRA01/04 cell line obtained from the SaiHongRui life sciences (NanJing, China)was cultured under the conditions as pLECs. Cells grown in the logarithmic phase were used for the experiment. Cells were seeded into 3.5 mm dishes and cultured with or without 5 ng/mL TGF-β2 for a period of 48 h in a serum-free medium when reaching 50% to 60% cell density.

### RNA sequencing

Total RNA was isolated from both the experimental and control groups using Trizol reagent (Thermo Scientific, USA,CAT#15596018). The total quantity and quality of RNA were evaluated using the RNA Nano 6000 Assay Kit in conjunction with the Bioanalyzer 2100 system (Agilent Technologies, CA, USA). To construct the sequencing library, one microgram of total RNA was treated using the NEBNext® Ultra™ RNA Library Prep Kit for Illumina® (NEB, USA), as per the manufacturer's instructions, and then analyzed on the Illumina NovaSeq 6000 platform. The DESeq2 R software package (1.20.0) was employed for the differential expression analysis between TGF-β2 treated and untreated pLECs. The resultant *P*-values were adjusted using Benjamini and Hochberg’s approach to manage the false discovery rate. A standard of Adj.*P*-value ≤ 0.05 and |log2(foldchange)|≥ 1 was used for screening differentially expressed genes (DEGs).

### Differential expression analysis and correlation analysis

We obtained 564 ferroptosis-related genes from the FerrDb database (http://www.zhounan.org/ferrdb/current/). We used R software to filtrate the ferroptosis-related DEGs in TGF-β2 treatment group and control groups in pLECs. To provide a more detailed understanding of the DEGs, we used the "ggplot2" and "pheatmap" packages to generate heat maps and a volcano plot. Additionally, a box plot was created using Graphpad Prism 9.0.

### Enrichment analysis of differentially expressed ferroptosis-related genes

We conducted Gene Ontology (GO) and Kyoto Encyclopedia of Genes and Genomes (KEGG) enrichment analyses of the DEGs using the clusterProfiler R package (version 3.8.1). An adjusted *P*-value of less than 0.05 indicated significant enrichment by the DEGs.

### PPI network

The protein–protein interaction (PPI) analysis of the DEGs was conducted utilizing the STRING database (https://string-db.org/). DEGs that were not related to any gene will be eliminated. The key subnetwork was extracted using Cytohubba plug-in in Cytoscape software (version 3.10.0), the 10 highest scoring genes, as determined by the MCC (Maximal Clique Centrality) algorithm, were considered as the hub genes.

### Quantitative real-time polymerase chain reaction (qRT-PCR)

We used SRA01/04 for validation and the RNA was extracted using the same methods used in RNA sequencing. The total RNA concentration was determined by spectrophotometry (NanoDrop; Thermo Fisher Scientific, USA), and cDNA was created with a reverse transcription kit (Yeasen, Shanghai, China CAT#11141). The expression levels were adjusted to match that of GAPDH. The primer sequences employed in this research can be found in the additional files.

## Statistics

All experimental data were statistically analyzed using the GraphPad Prism 9.0 software. Results are presented as the mean ± SD, based on a minimum of three independent replications. A Student's t-test was applied for comparing two independent samples (using the Holm-Bohemiak method for multiple comparisons), and an adjusted *P*-value of less than 0.05 was deemed to be statistically significant.

## Results

### Identification of genes in pLECs with or without TGF-β2 treated

**Fig. 1:**
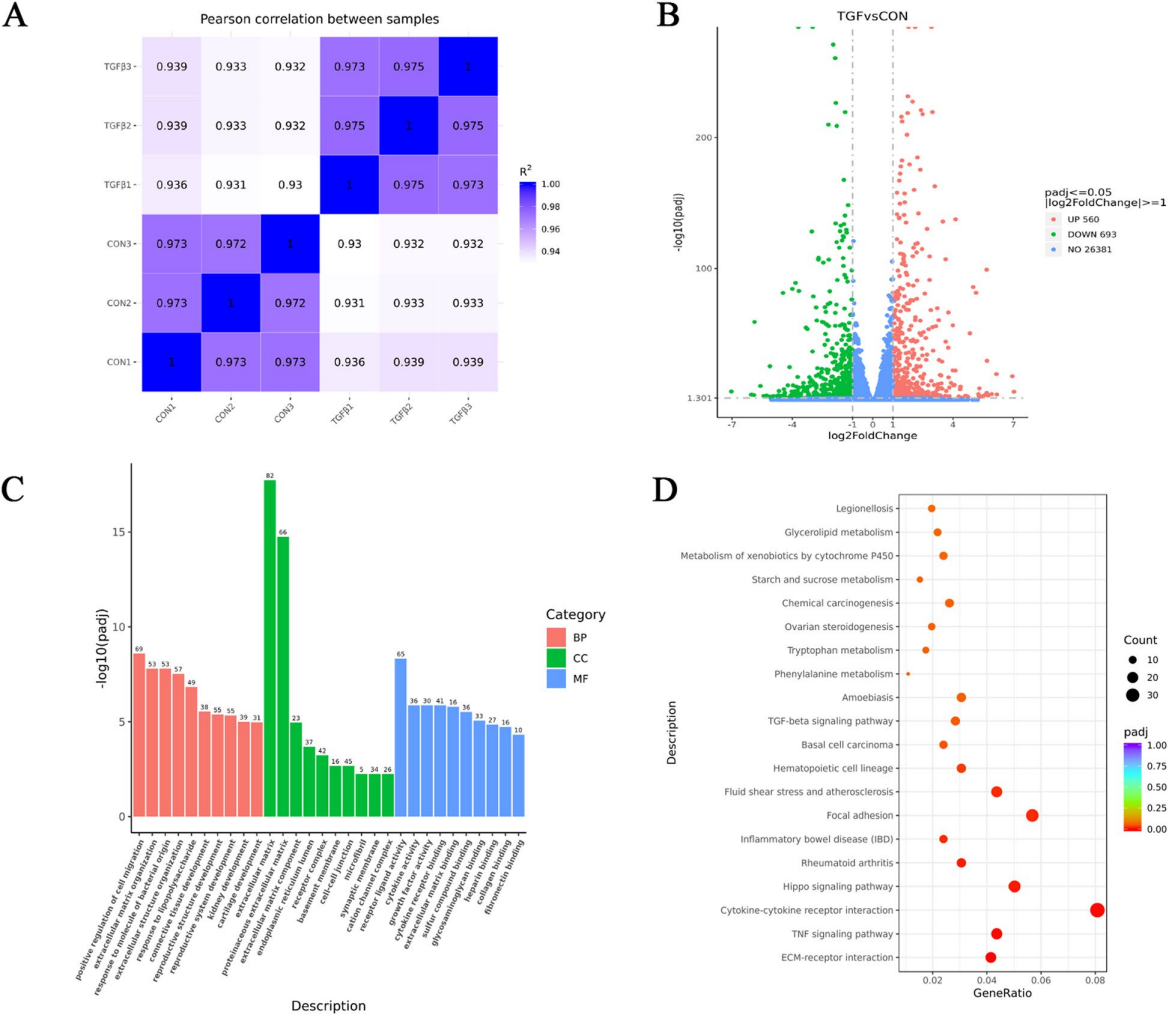
Identification of genes in pLECs with or without TGF-β2 treated. **A** Pearson correlation analysis in TGFβ2-treated or -untreated pLECs. **B** Volcano plot of 1253 DEGs. **C** GO enrichment analysis of all DEGs. **D** TOP 20 KEGG enrichment analysis of all DEGs

In our study, we used TGF-β2 to treat pLECs to simulate the EMT process in the formation of fibrotic PCO. Firstly, the correlation of gene expression between groups was detected by Pearson correlation analysis, The results (Fig. 1A) revealed that the squared Pearson correlation coefficient (R2) of each sample in the two groups is greater than 0.92, indicating excellent experimental repeatability. We then identified the differential genes in TGF-β2-treated or -untreated pLECs, and 1253 DEGs were found in the two groups (Fig. 1B), in which 560 genes were up-regulated and 693 genes were down-regulated (log2|fold-change|≥ 1; Adj.*P*-value ≤ 0.05). All the DEGs were subsequently used for GO and KEGG analysis. Figure 1C presents the top ten biological processes (BPs), molecular functions (MFs) and cellular components (CCs), most of which were pathways associated with EMT. For instance, positive regulation of cell migration, extracellular matrix, collagen binding and fibronectin binding. Similarly, the results of KEGG pathways displayed that genes were primarily associated with cytokine-cytokine receptor interaction, focal adhesion and hippo signaling pathway (Fig. 1D).

### Identification of differentially expressed ferroptosis-related genes

**Fig. 2:**
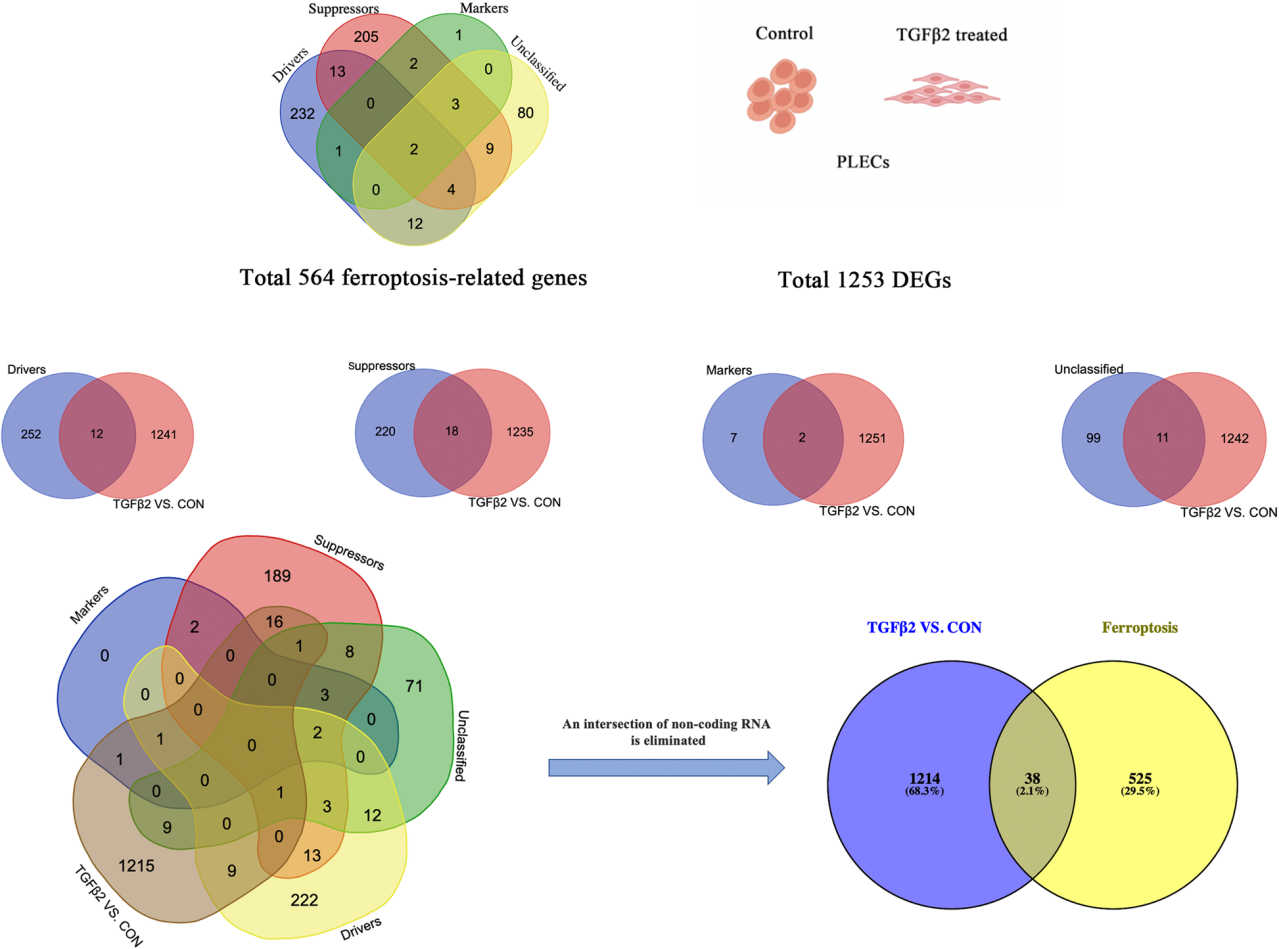
Screening process for genes associated with ferroptosis

**Fig. 3:**
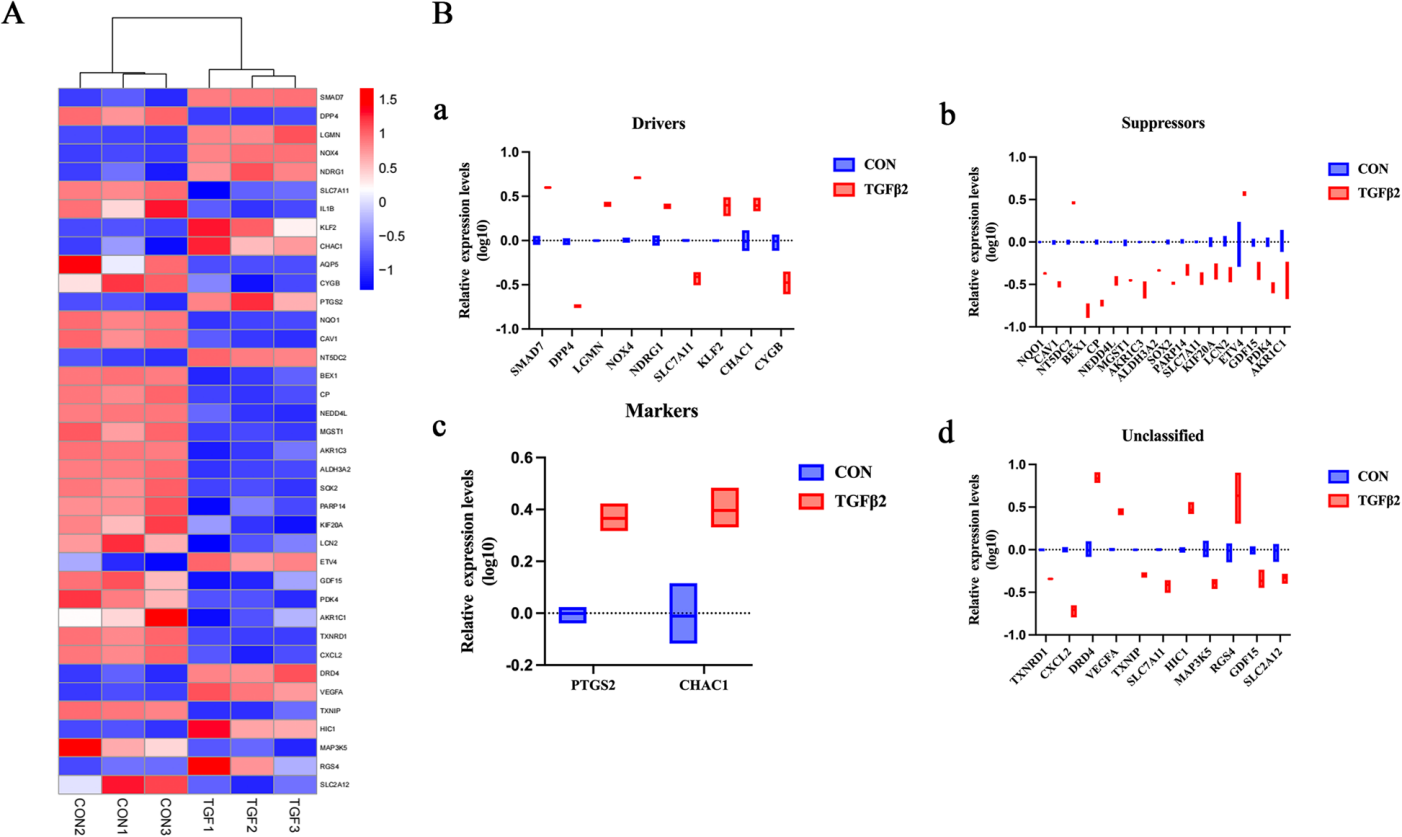
Identification of 38 differentially expressed ferroptosis-related genes. **A** Heatmap of 38 differentially expressed ferroptosis-related genes. **B** Box line plot of 38 differentially expressed ferroptosis-related genes. a, Box line plot of ferroptosis drivers, b, Box line plot of ferroptosis suppressors, c, Box line plot of ferroptosis markers, d, Box line plot of genes with unclassified roles in ferroptosis. Blue represents control group and red represents TGF-β2 treated group

We obtained 564 genes associated with ferroptosis including drivers, suppressors, markers and unclassified from the FerrDb Database (http://www.zhounan.org/ferrdb/current/). Then, we merged the 1253 differential genes screened by sequencing results with the genes contained in FerrDb database, identified 12 ferroptosis drivers (Table 1), 18 suppressors (Table 2), 2 markers (Table 3) and 11 unclassified genes (Table 4).After, excluding 3 duplicate genes and one non-coding RNA, we ended up with a collection of 38 genes related to ferroptosis. Out of these, 13 genes demonstrated an up-regulation, while 25 showed a down-regulation (Refer to Fig. 2). Figure 3A provides a heat map representation of the 38 variably expressed ferroptosis-related genes when comparing the control with TGF-β2 treated pLECs. Furthermore, a box line plot displaying the degree of differential expression of the 38 genes across four different categories of ferroptosis-related genes between two groups is shown in Fig. 3B.

**Table 1:** The 11 differentially expressed Ferroptosis drivers-related genes in TGF-β2-treated or -untreated pLECs

Gene name	Gene description	Log2FoldChange	Change	*P*-value	Adj. *P*-value
SMAD7	SMAD family member 7	1.992841	Up	1.49E-112	3.84E-110
DPP4	Dipeptidyl peptidase 4	-2.462277	Down	1.27E-107	2.87E-105
LGMN	Legumain	1.337992	Up	2.63E-97	5.26E-95
NOX4	NADPH oxidase 4	2.363958	Up	5.57E-63	5.63E-61
NDRG1	N-myc downstream regulated 1	1.27335	Up	3.79E-40	2.27E-38
SLC7A11	Solute carrier family 7 member 1	-1.345309	Down	1.43E-18	3.48E-17
IL1B	Interleukin 1 beta	-4.420076	Down	8.24E-10	1.02E-08
KLF2	Kruppel like factor 2	1.355188	Up	4.35E-08	4.48E-07
CHAC1	ChaC glutathione specific gammaglutamylcyclotransferase 1	1.332998	Up	2.59E-07	2.42E-06
AQP5	Aquaporin 5	-6.051828	Down	1.72E-05	1.24E-04
CYGB	Cytoglobin	-1.539514	Down	1.95E-05	1.39E-04

**Table 2:** The 18 differentially expressed Ferroptosis suppressors-related genes in TGF-β2-treated or -untreated pLECs

Gene name	Gene description	Log2FoldChange	Change	*P*-value	Adj. *P*-value
NQO1	NAD(P)H quinone dehydrogenase 1	-1.225004	Down	7.59E-152	3.90E-149
CAV1	Caveolin 1	-1.671334	Down	4.42E-119	1.26E-116
NT5DC2	5’-nucleotidase domain containing 2	1.52302	Up	1.64E-110	3.99E-108
BEX1	Brain expressed X-linked 1	-2.701982	Down	6.52E-110	1.54E-107
CP	Ceruloplasmin	-2.3792	Down	2.07E-73	2.59E-71
NEDD4L	Neural precursor cell expressed, developmentally down-regulated 4-like, E3 ubiquitin protein ligase	-1.547678	Down	2.87E-48	2.01E-46
MGST1	Microsomal glutathione S-transferase 1	-1.502248	Down	1.63E-39	9.54E-38
AKR1C3	Aldo–keto reductase family 1 member C3	-1.883406	Down	3.61E-35	1.82E-33
ALDH3A2	Aldehyde dehydrogenase 3 family member A2	-1.111655	Down	3.69E-35	1.86E-33
SOX2	SRY-box 2	-1.614247	Down	1.42E-29	5.77E-28
PARP14	Poly(ADP-ribose) polymerase family member 14	-1.069349	Down	1.22E-26	4.32E-25
SLC7A11	Solute carrier family 7 member 11	-1.345309	Down	1.43E-18	3.48E-17
KIF20A	Kinesin family member 20A	-1.181047	Down	3.10E-08	3.25E-07
LCN2	Lipocalin 2	-1.231111	Down	7.60E-08	7.57E-07
ETV4	ETS variant 4	1.891098	Up	5.31E-06	4.15E-05
GDF15	Growth differentiation factor 15	-1.17786	Down	1.29E-04	7.91E-04
PDK4	Pyruvate dehydrogenase kinase 4	-1.713539	Down	7.07E-04	3.67E-03
AKR1C1	Aldo–keto reductase family 1 member C1	-1.357027	Down	2.53E-03	1.14E-02

**Table 3:** The 2 differentially expressed Ferroptosis markers-related genes in TGF-β2-treated or -untreated pLECs

Gene name	Gene description	Log2FoldChange	Change	*P*-value	Adj. *P*-value
PTGS2	prostaglandin-endoperoxide synthase 2	1.221535	Up	1.93E-07	1.83E-06
CHAC1	ChaC glutathione specific gamma-glutamylcyclotransferase 1	1.332998	Up	2.59E-07	2.42E-06

**Table 4:** The 11 differentially expressed Ferroptosis unclassified genes in TGF-β2-treated or -untreated pLECs

Gene name	Gene description	Log2FoldChange	Change	*P*-value	Adj. *P*-value
TXNRD1	Thioredoxin reductase 1	-1.137716	Down	4.41E-119	1.26E-116
CXCL2	CXC motif chemokine ligand 2	-2.324465	Down	2.62E-60	2.50E-58
DRD4	Dopamine receptor D4	2.787184	Up	2.21E-56	1.88E-54
VEGFA	Vascular endothelial growth factor A	1.480892	Up	4.20E-53	3.37E-51
TXNIP	Thioredoxin interacting protein	-1.015813	Down	8.56E-34	4.12E-32
SLC7A11	Solute carrier family 7 member 11	-1.345309	Down	1.43E-18	3.48E-17
HIC1	HIC ZBTB transcriptional repressor 1	1.569275	Up	7.26E-16	1.47E-14
MAP3K5	Mitogen-activated protein kinase kinase kinase 5	-1.293284	Down	1.37E-07	1.31E-06
RGS4	Regulator of G protein signaling 4	2.318594	Up	3.19E-06	2.57E-05
GDF15	Growth differentiation factor 15	-1.17786	Down	1.29E-04	7.91E-04
SLC2A12	Solute carrier family 2 member 12	-1.078542	Down	4.44E-03	1.87E-02

### Enrichment analysis of functions and pathways for the differentially expressed genes associated with ferroptosis.

**Fig. 4:**
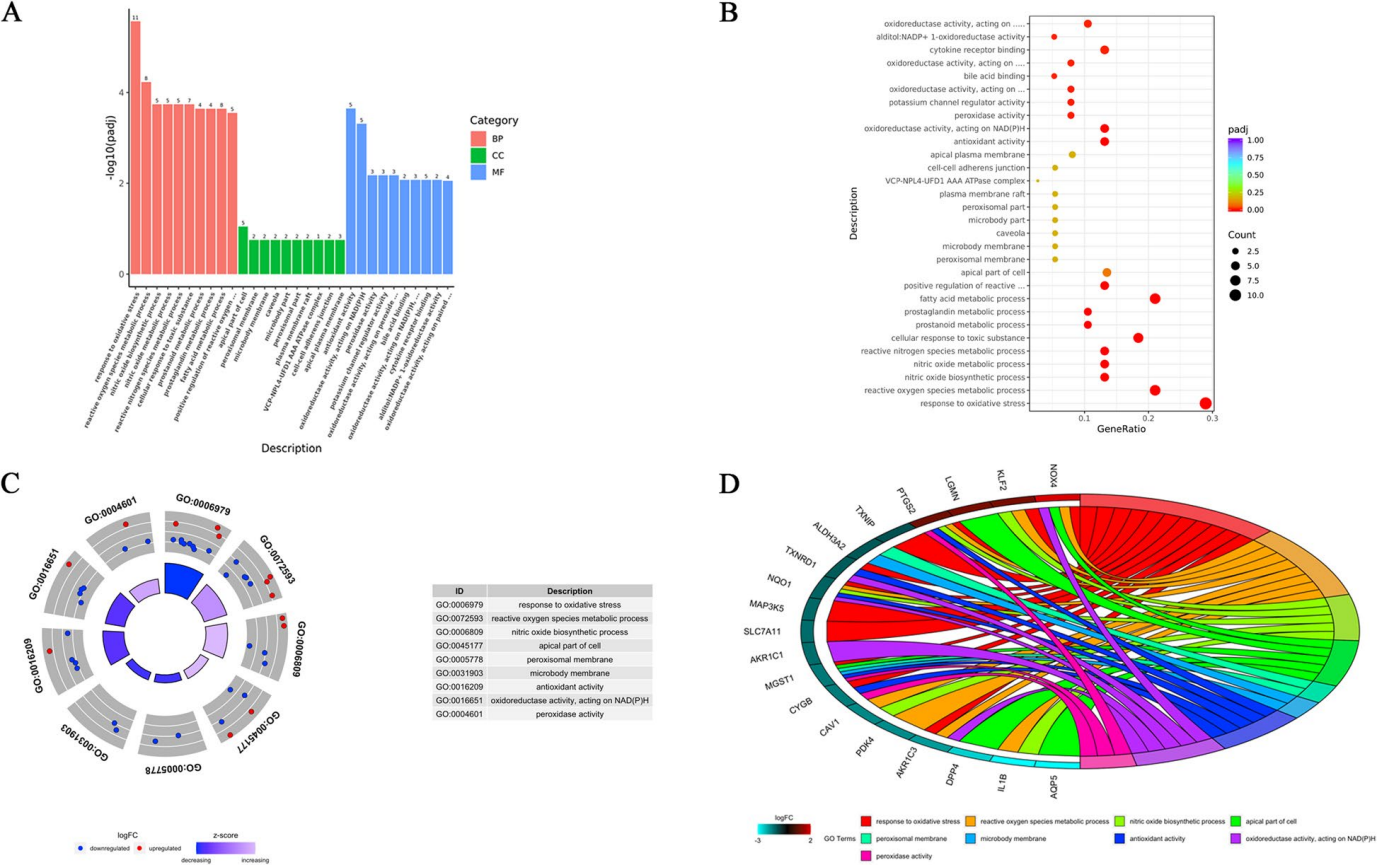
Go enrichment analysis of 38 differentially expressed ferroptosis-related genes. **A** Barplot of the top 10 in GO analysis (BPs, CCs and MFs respectively). **B** Dotplot of the top 10 in GO analysis (BPs, CCs and MFs respectively). **C** GOCircle of the top 3 in GO analysis (BPs, CCs and MFs respectively). **D** GOChord of enriched GO process, it shows the association between DEGs and the top 3 function in BPs, CCs and MFs

**Fig. 5:**
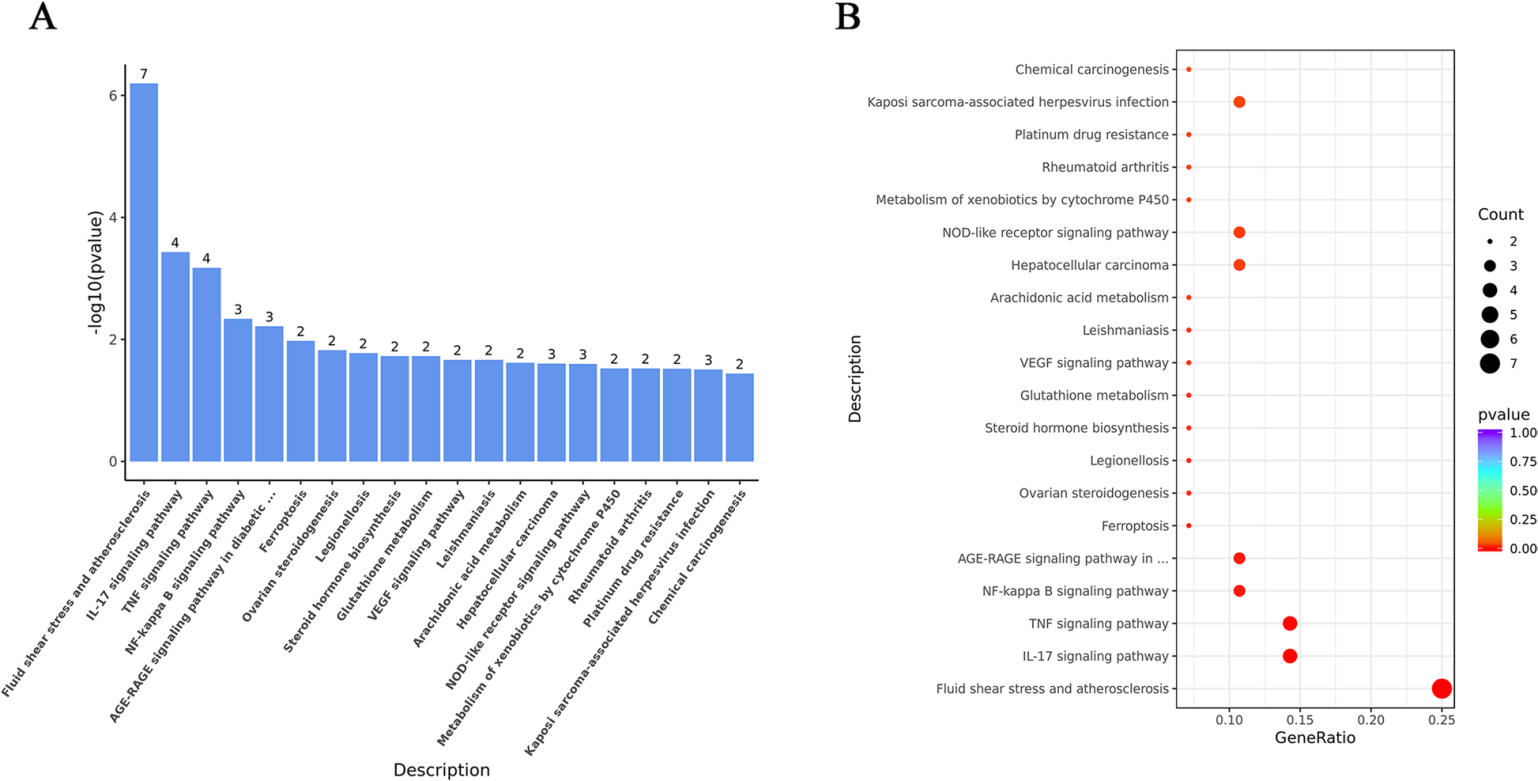
KEGG enrichment analysis of 38 differentially expressed ferroptosis-related genes. **A** Barplot of the top 10 KEGG enrichment pathways. **B** Dotplot of the top 20 KEGG enrichment pathways

To explore the potential functions of ferroptosis related differential genes from the biological function level, we conducted GO and KEGG analyses, as shown in Fig. 4. The GO analysis revealed significant enrichment of the DEGs in 754 BPs, 28 CCs, and 80 MFs (*P*-value < 0.05). The top 10 biological processes for each of the three categories are depicted in Fig. 4A, B. Notably, these processes included response to oxidative stress, reactive oxygen species metabolic process, and nitric oxide biosynthetic process (BPs); apical part of cell, peroxisomal membrane, and microbody membrane (CCs); antioxidant activity, oxidoreductase activity acting on NAD(P)H, and peroxidase activity (MFs) (See Fig. 4C, D). Additionally, 19 common genes participated in these key processes (Refer to Table 5). We also conducted a KEGG analysis for these 38 genes, revealing their primary involvement in fluid shear stress and atherosclerosis, IL-17 signaling, and TNF signaling pathways (See Fig. 5).

**Table 5:** The prominent process in GO enrichment analysis

Category	GOID	Description	GeneRatio	*P* value	geneName	Common genes
BP	GO:0006979	Response to oxidative stress	11/38	1.53E-09	NOX4/SLC7A11/KLF2/CYGB /PTGS2/NQO1/MGST1/AKR1C3 /TXNRD1/TXNIP/MAP3K5	NOX4/SLC7A11/KLF2 /CYGB/PTGS2/NQO1 /MGST1/AKR1C3/TXNRD1 /TXNIP/MAP3K5/PDK4 /IL1B/CAV1/DPP4 /LGMN/AQP5/ALDH3A2 /AKR1C1
BP	GO:0072593	Reactive oxygen species metabolic process	8/38	6.63E-08	NOX4/IL1B/KLF2/PTGS2 /NQO1/CAV1/ AKR1C3/PDK4	
BP	GO:0006809	Nitric oxide biosynthetic process	5/38	3.66E-07	IL1B/KLF2/PTGS 2/NQO1/CAV1	
CC	GO:0045177	Apical part of cell	5/37	0.000818234	DPP4/LGMN/NOX4 /AQP5/MGST1	
CC	GO:0005778	Peroxisomal membrane	2/37	0.006112096	MGST1/ALDH3A2	
CC	GO:0031903	Microbody membrane	2/37	0.006112096	MGST1/ALDH3A2	
MF	GO:0016209	Antioxidant activity	5/38	1.07E-06	CYGB/PTGS2/NQO1 /MGST1/TXNRD1	
MF	GO:0016651	Oxidoreductase activity, acting on NAD(P)H	5/38	4.66E-06	NOX4/NQO1/AKR1C3 /AKR1C1/TXNRD1	
MF	GO:0004601	Peroxidase activity	3/38	0.000122681	CYGB/PTGS2/MGST1

### PPI network and recognition of hub genes

**Fig. 6:**
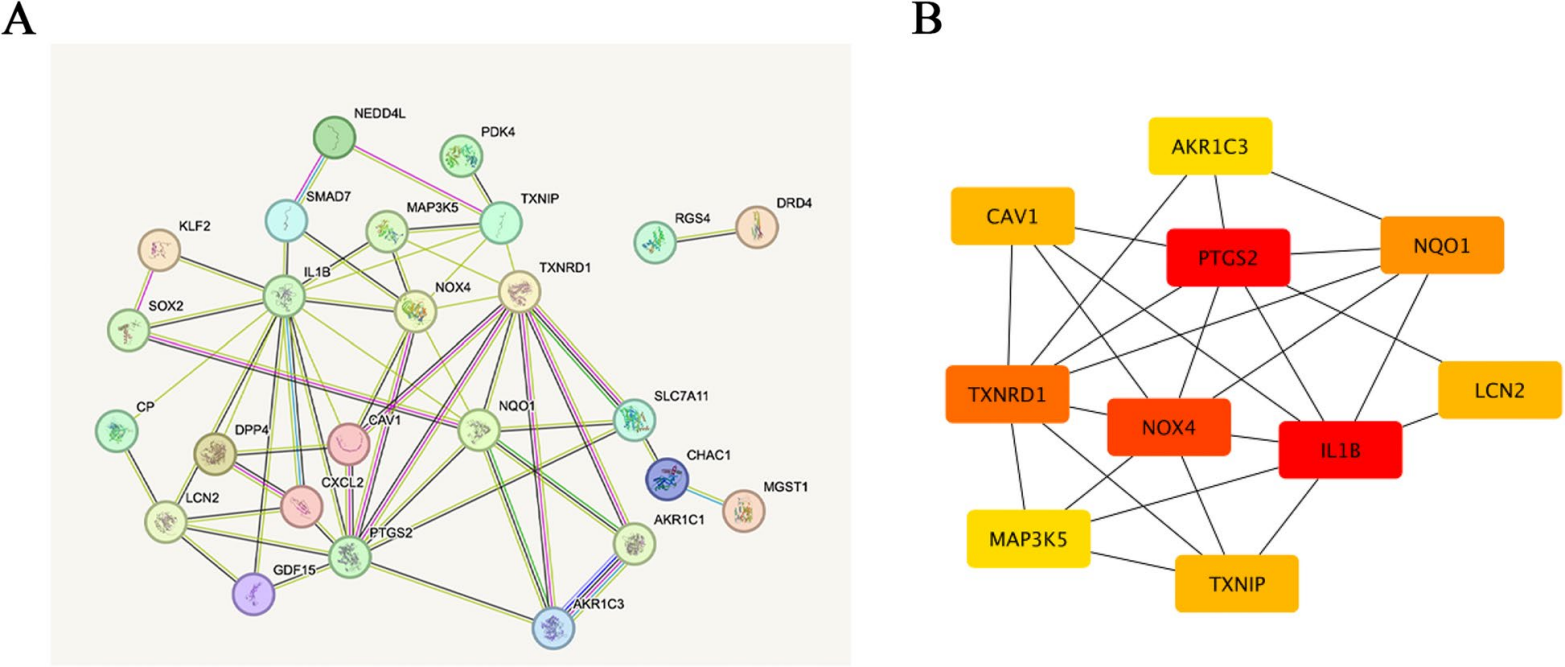
PPI network and Recognition of hub genes. **A** Each node represents a protein, and the lines represent an association between proteins. **B** Among the 38 ferroptosis-related genes, the top 10 hub genes were identified by using Cytoscape

To understand protein-to-protein interactions among selected ferroptosis-related genes, we used STRING database to construct PPI network (Fig. 6A). During analysis, we found 25 nodes and 54 interaction pairs, removing those proteins not associated with any other protein. In addition, we used MCC algorithm in Cytoscape software to obtain the 10 top-scoring genes (Fig. 6B). Among them, PTGS2 and NOX4 were found to be upregulated, while the other genes were downregulated (Table 6). These 10 ferroptosis-related genes were considered to be most closely associated the induced fibrosis model and warrant further investigation in the context of the onset and progression of PCO in the future.

**Table 6:** Top 10 in PPI network ranked by MCC algorithm

Rank	Gene name	Description	MCC score	Change
1	PTGS2	Prostaglandin-endoperoxide synthase 2	48	Up
2	IL1B	Interleukin 1 beta	42	Down
3	NOX4	NADPH oxidase 4	38	Up
4	TXNRD1	Thioredoxin reductase 1	36	Down
5	NQO1	NAD(P)H quinone dehydrogenase 1	32	Down
6	CAV1	Caveolin 1	14	Down
7	LCN2	Lipocalin 2	14	Down
8	TXNIP	Thioredoxin interacting protein	14	Down
9	AKR1C3	Aldo–keto reductase family 1 member C3	12	Down
10	MAP3K5	Mitogen-activated protein kinase kinase kinase 5	12	Down

### Validation of differentially expressed ferroptosis-related genes using qRT-PCR

**Fig. 7:**
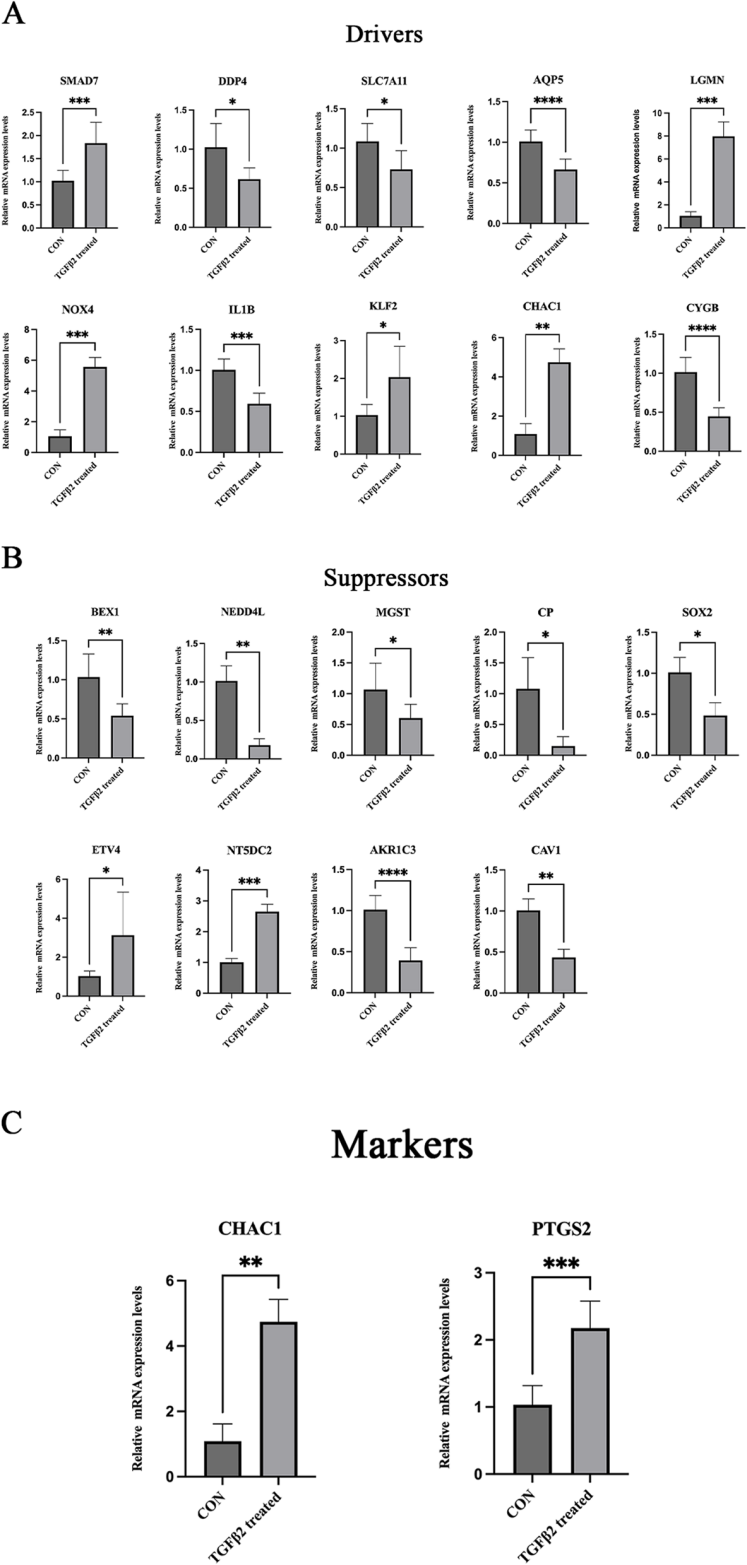
Validation of the expression trend of differentially expressed ferroptosis-related genes. **A** Validation of ferroptosis divers. **B** Validation of ferroptosis suppressors. **C** Validation of ferroptosis markers. *Adj.*P*-value < 0.05, **Adj.*P*-value < 0.01, ***Adj.*P*-value < 0.001, **** Adj.*P*-value < 0.0001, *n* > 3

SRA01/04 underwent in vitro culture with TGF-β2 (5 ng/ml) for 48 h to mimic the EMT process post-cataract surgery. The top 10 ferroptosis-related genes with the highest fold-change values, including drivers, suppressors, and markers, were confirmed using qRT-PCR. All results were statistically significant (with an adjusted *P*-value less than 0.05), and the trends aligned with the sequencing outcomes(Fig. 7). However, PDK4 was expressed at very low levels in both the control and TGF-β2 treatment groups and was omitted from the analysis.

## Discussion

PCO significantly impact the long-term visual quality after cataract surgery [18]. If LECs fail to proliferate and differentiate into lens fibers, it will transdifferentiate into mesenchymal cells, which is the process of EMT [20]. EMT can be triggered by an inflammatory response [19], such as the ocular response after cataract surgery [21], in which damaged eye tissue releases chemokines to attract immune system cells to clear the damaged tissue and promote tissue repair. During the wound healing process, a multitude of cytokines, including tumor necrosis factor alpha (TNF- α), interleukin 1β (IL-1β), and prostaglandins, play pivotal roles in modulating tissue response [18, 22]. This healing response influences cell proliferation, migration, and differentiation, leading to an enhanced expression of EMT markers like α-smooth muscle actin (α-SMA), Vimentin, Fibronectin (FN), and Collagen type I (Col I) [23], Conversely, the expression of epithelial markers such as E-Cadherin (E-Cad) is reduced, resulting in the formation of PCO.

TGF-β serves as a key factor in the EMT process, with TGF-β2 being the predominant isomer present in human aqueous humor. Despite the high levels of TGF-β present in the aqueous humor, most of it remains in an inactive state. [24]. When inactive TGF-β is activated by proteolytic cleavage or other mechanisms after eye injury or surgery [17, 25] and released from inhibitory binding proteins [26], the initiation of fibrotic PCO can occur through both SMAD-dependent and SMAD-independent pathways. It is widely believed that active TGF-β [17, 18]can cause fibrotic PCO and ASC. Cultured LECs undergo EMT upon treatment with exogenous active TGF-β and the fibrotic plaques found in ASC exhibit high levels of phosphorylated Smad2/3, a major effector of TGFβ signaling [27, 28]. Furthermore, the treatment of lenses with active TGF-β either in culture [29] or via transgenic overexpression [30, 31], leads to ASCs expressing myofibroblast markers as a result of EMT process of LECs. Therefore, in our study, we used TGF-β2 to stimulate pLECs extracted from the anterior capsule, obtained during pediatric cataract surgery, to mimic the EMT in PCO. Based on literature search, this is the first time that pLECs of human origin have been cultured for RNA sequencing analysis.

Ferroptosis, a type of cell death triggered by oxidative stress, is deeply connected to cell metabolism [14].Simultaneously, oxidative stress triggers inflammation in lens cells, which escalates cytokine expression, EMT, and the advancement of PCO [32]. As a result, we aimed to explore the significance of ferroptosis in the progression of PCO. At present, studies on the role of ferroptosis in cataract mainly focus on the pathogenesis of ARC. Wei et al. [33]first put forward that LECs exhibit high sensitivity to ferroptosis, their research discovered that even minimal concentrations of system X inhibitor Erastin and glutathione peroxidase 4 (GPX4) inhibitor RSL3 significantly triggered ferroptosis in human LECs (FHL124) and mouse lens epithelium. A significant reduction of intracellular glutathione (GSH) in human and mouse LECs notably increases their sensitivity to ferroptosis, especially in response to RSL3 stimulation. Another study identified ferroptosis as the primary pathological process in LECs of ARC patients, a finding also observed in aged mice [34].They demonstrated that LECs ferroptosis is regulated by Nrf2 downregulation and abnormal increase in GSK-3β expression. Furthermore, UVB-induced ferroptosis was found to lead to ARC by downregulating the expression of SIRT6 [35], Additionally, the subconjunctival administration of Melatonin was found to mitigate and delay cataract progression by triggering the SIRT6/p-Nrf2/GPX4 and SIRT6/NCOA4/FTH1 signaling pathways, respectively. All these studies imply a role for ferroptosis in cataract formation, but the effect of ferroptosis in PCO remains unexplored.

The occurrence of EMT signifies the loss of the original characteristics of epithelial cells, including cell polarity, epithelial markers, cell–cell adhesion, and tight junctions. Consequently, they acquire traits resembling mesenchymal cells [36]. There are currently numerous studies exploring the interconnections between EMT and ferroptosis in various fields. In our study, we identified 38 potential ferroptosis-related genes in TGF-β2-treated or -untreated pLECs, and 10 hub genes were further screened, including PTGS2, NOX4, IL1B, TXNRD1, LCN2, AKR1C3, CAV1, NQO1, MAP3K5, TXNIP. The role of some genes in the progress of PCO has been extensively reported, Chandler et al. [37] provided evidence that the application of COX-2 inhibitors in vivo could reduce migration and proliferation while increasing cellular apoptosis to prevent PCO. Research also indicates that ferroptosis can directly increase the expression of COX2 and enhance the secretion of inflammatory signals. Inflammatory compounds stimulate late-stage transcription factors such as Smads, NF-κB, STAT3, Snail, Twist, and ZEB promoting EMT [38]. Studies has shown that TGF-β promotes the expression of NOX4 in rat LECs and inhibition of NOX4 can block the EMT process [39, 40], also DAS et al. [41] proposed that the deletion of NOX4 is in both in vitro and in vivo only a partial repeal of the EMT response, which it was clear that other sources of reactive oxygen species (ROS) have an impact in the EMT process of LECs. Extracapsular extraction of mouse lens model is used to the study of PCO pathogenesis [42–44]. Immunolocalization revealed that LCN2 protein levels were upregulated in LECs within 1–6 h post extracapsular extraction of mouse lens and peak at 24 h as our previous study showed [45],which sets the foundation for the subsequent initiation of EMT. Moreover, A study reported that Circ-POLR3A enhances TGF-β2-induced viability, migration, and invasion in SRA01/04 through miR-31/TXNIP signaling pathway [46]. But in fact, the crosstalk between ferroptosis and EMT in LECs has not been reported or investigated yet.

How ferroptosis is involved in the EMT process in LECs? Firstly, we hypothesized that LECs that undergo EMT become more sensitive to ferroptosis. Studies have reported that the presence of tight junctions and epithelial-related markers in epithelial cells prevents ferroptosis by promoting cell-to-cell adhesion. In contrast, cancer cells in a mesenchymal state exhibit reduced cell-to-cell contacts and acquire mesenchymal properties, making them more susceptible to ferroptosis [47].Secondly, the loss of cell–cell adhesion increases the vulnerability of cells to lipid peroxidation, leading to ferroptosis [48], it has been observed that overexpressed enzymes such as ACSLs and SCD1, which are involved in lipid metabolism and increased unsaturation index, induce EMT and increase the migration/invasion of colorectal cancer cells [49]. Furthermore, after TGF-β1 induces EMT in melanoma cells, the expression of ferritin heavy chain 1 (FTH1) decreases, leading to an increase in labile iron pool (LIP) and intracellular ROS generation. The accumulation of iron and the increase in oxidative stress are key events in ferroptosis [50].At the same time, the occurrence of ferroptosis will also aggravate the EMT process. In vivo, TGF-β1 triggers the generation of ROS in epithelial cells, suppresses the activity of antioxidant enzymes, and disrupts cellular redox status. Subsequently, ROS either induces or activates TGF-β1, contributing to the development of pulmonary fibrosis. This reciprocal interaction establishes a detrimental cycle [50, 51]. In the future, we will explore the concrete mechanism of ferroptosis in the EMT process of LECs.

Our study also has its limitations, relying solely on semi-quantitative methods such as RNA sequencing is not sufficient. In the future, further investigations are needed to uncover the underlying mechanisms of the interplay between ferroptosis and EMT. This not only enhances our understanding of the pathogenesis of PCO but also provides new strategies for its treatment. Reversing or inhibiting EMT, as well as regulating genes and signaling pathways associated with ferroptosis, may bring breakthroughs in PCO therapy.

## Conclusions

We initiated our investigation by employing qRT-PCR to validate the expression of 20 ferroptosis-associated genes in human lens epithelial cell lines (SRA01/04) at the RNA level. Our finding presents the first identification of ferroptosis related genes in primary human lens epithelial cells subjected to TGF-β2 stimulation. These findings may enhance our understanding of the molecular mechanisms of PCO, but its specific pathogenesis and regulation process still require in-depth investigation. We hope that ferroptosis or its related genes can be used as new targets for the study on onset, and progression of PCO, thereby offering a novel approach for the treatment of PCO.

### Supplementary Information

**Supplementary Material 1. **

**Supplementary Material 2. **

## Data Availability

The datasets used and analyzed during the current study are available from the corresponding author on reasonable request.

## Abbreviations

PCO: Posterior capsular opacification
TGFβ: Transforming growth factor beta
RNA-seq: RNA sequencing
pLECs: Primary lens epithelial cells
DEGs: Differentially expressed genes
GO: Gene Ontology
KEGG: Kyoto Encyclopedia of Genes and Genomes
qRT-PCR: Quantitative real-time polymerase chain reaction
EMT: Epithelial-mesenchymal transition
LECs: Lens epithelial cells
IOL: Intraocular lens
ARC: Age-related cataract
DMEM: Dulbecco's Modified Eagle Medium
PPI: Protein–protein interaction
BPs: Biological processes
MFs: Molecular functions
CCs: Cellular components
TNF-α: Tumor necrosis factor alpha
IL-1β: Interleukin 1β
α-SMA: α-Smooth muscle actin
FN: Fibronectin
Col I: Collagen type I
E-Cad: E-Cadherin
ASC: Anterior subcapsular cataract
GPX4: Glutathione peroxidase 4
GSH: Glutathione
FTH1: Ferritin heavy chain 1
LIP: Labile iron pool
ROS: Intracellular reactive oxygen species
