# Supplementary figures and images for: Transcriptome exploration of ferroptosis-related genes in TGFβ- induced lens epithelial to mesenchymal transition during posterior capsular opacification development
Source: BMC Genomics. 2024 Apr 9;25:352. doi: 10.1186/s12864-024-10244-y (PMC11003017; doi:10.1186/s12864-024-10244-y)

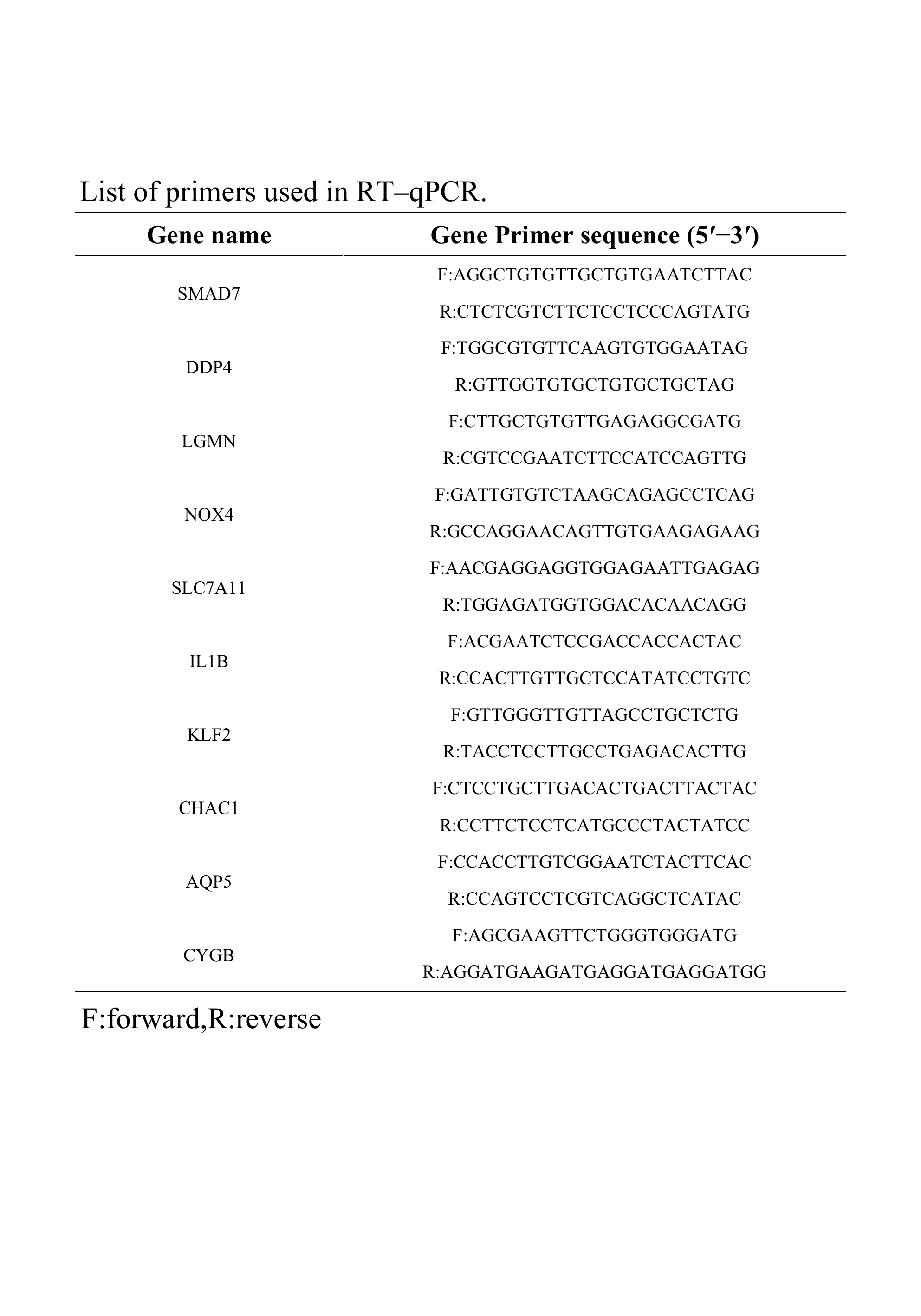

Supplement: Supplementary file 1 — Supplementary Material 1. [file 12864_2024_10244_MOESM1_ESM.tif]

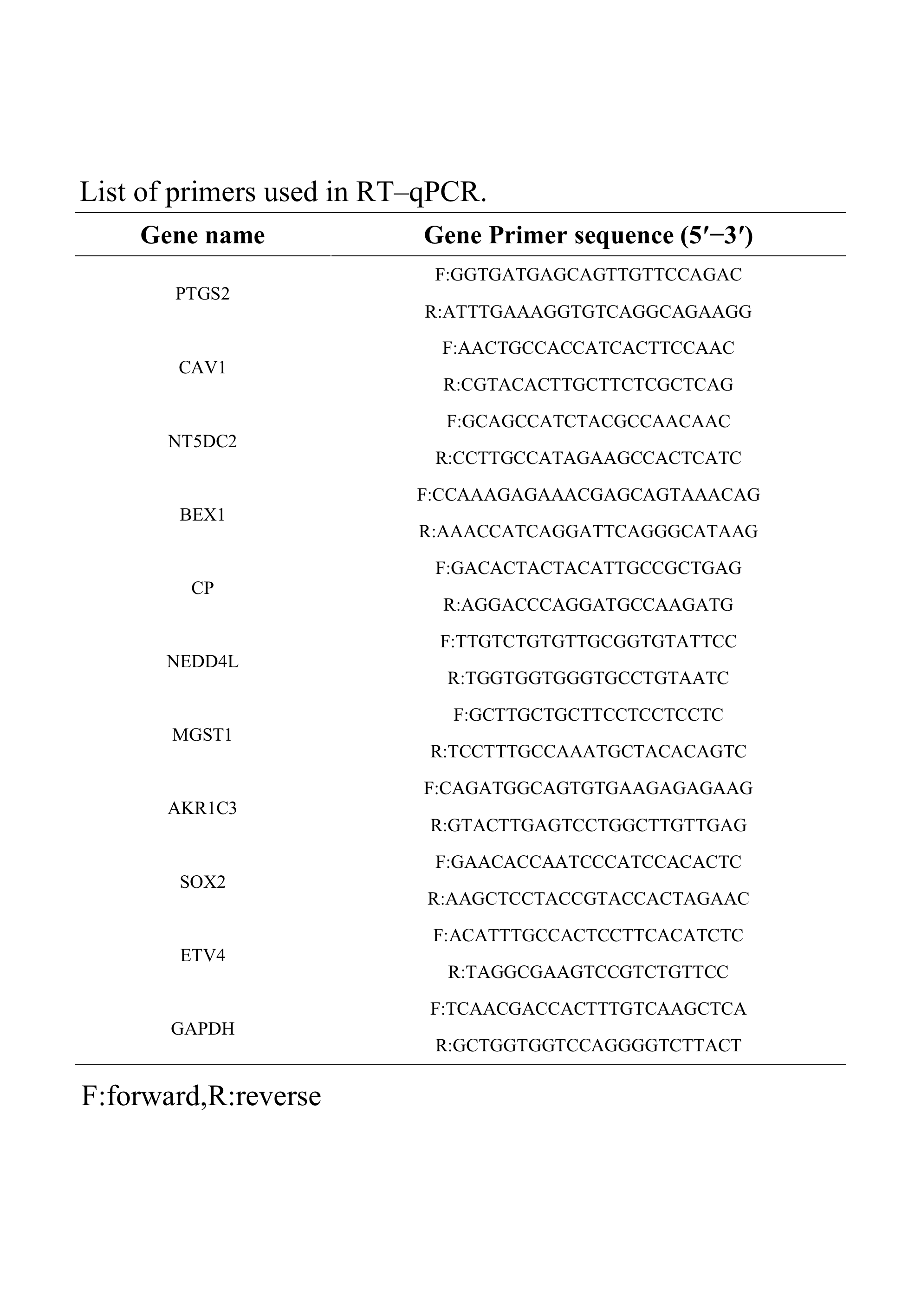

Supplement: Supplementary file 2 — Supplementary Material 2. [file 12864_2024_10244_MOESM2_ESM.tif]
